# Effectiveness of an individual acceptance and commitment therapy for smoking cessation, delivered face-to-face and by telephone to adults recruited in primary health care settings: a randomized controlled trial

**DOI:** 10.1186/s12889-020-09820-0

**Published:** 2020-11-16

**Authors:** Yim Wah Mak, Doris Y. P. Leung, Alice Yuen Loke

**Affiliations:** School of Nursing, The Hong Kong Polytechnic University, Yuk Choi Road, Hung Hom, Kowloon, Hong Kong, Special Administrative Region of China

**Keywords:** Smoking cessation program, Acceptance and commitment therapy (ACT), Face-to-face, Telephone, Primary health care, Randomized controlled trial (RCT)

## Abstract

**Background:**

The aim of this study was to examine the effectiveness of delivering an individual Acceptance and Commitment Therapy (ACT) for smoking cessation among a Chinese population.

**Methods:**

Participants were recruited from six primary health care centers. A total of 144 were eligible to take part in the study and agreed to be randomized to the intervention (ACT) group (*n* = 70) and control group (*n* = 74), respectively. Both groups received self-help materials on smoking cessation. The ACT group also underwent an initial face-to-face session and two telephone ACT sessions at 1 week and 1 month following the first session. They were re-contacted through telephone follow-ups at 3, 6, and 12 months by research assistants. The primary outcome was self-reported 7-day point-prevalence abstinence at the 12-month follow-up session. Other outcomes included biochemically validated quitting, quitting attempts, the intention to quit, the self-perception of quitting, and psychological flexibility.

**Results:**

There was no significant difference in the self-reported 7-day point prevalence quit rate at the 12-month follow-up between the intervention group (24.3%) and the control group (21.6%) (risk ratio = 1.12; 95%CI = (0.62, 2.05); *p* = 0.704). Greater improvements in secondary outcomes from baseline to the 12-month follow-up were observed in the ACT group than in the control group, including a forward progression in the participants’ readiness to quit smoking (*p* = 0.014) and increased psychological flexibility (*p* = 0.022).

**Conclusions:**

This study is the first evidence of a randomized-controlled trial on the adoption of an individual ACT for smoking cessation, delivered initially in primary health care settings and subsequently by telephone within a Chinese population. The present study found that the brief ACT intervention could not produce a significant quit rate but was promising in terms of bringing about cognitive changes, including greater psychological flexibility, and more confidence about quitting, when compared to the use of self-help materials only among the general population.

**Trial registration:**

This trial was registered prospectively with the U.S. National Library of Medicine: (NCT01652508) on 26th July 2012.

## Background

With over 1.1 billion smokers worldwide [1], smoking-related deaths such as cancer and respiratory and vascular diseases are predicted to reach 10 million by 2025 [2]. The population of China is one the world’s greatest consumers of tobacco [3]. In 2010, the prevalence of smoking among adults in China was 28.1%, at 52.9% for men and 2.4% for women [4], while the prevalence in Hong Kong in 2018 was a relatively low 10%, with 18.1% for men and 2.7% for women [5]. However, similar to global figures [2], half of all deaths among smokers aged 65 years or older in Hong Kong were caused by smoking [6]. Access to effective smoking cessation programs is, therefore, a major public need. A range of behavioral interventions has been shown to be effective for achieving smoking cessation [7, 8]. Given cultural similarities between Hong Kong and mainland China, a smoking cessation intervention that proves to be effective in Hong Kong could likely be beneficially implemented in mainland China. Previous studies have found that it is feasible to approach smokers through community-based health care settings in Hong Kong [9] and also in mainland China [10], even though the smokers were not there to seek smoking cessation services. However, many of the individuals in community health care settings who were approached were unable to take part in smoking cessation programs due to a lack of time [9].

Since a telephone-based intervention makes smoking cessation counseling more easily accessible [11] and has been found to be feasible and effective in Hong Kong [12], mainland China, and many countries [13–15], recruiting smokers to join a cessation program at health services clinics and providing follow-up sessions by phone is a feasible approach [12]. Telephone-based smoking cessation interventions have been adopted for many years in Hong Kong [16] and in other countries [15]. The weighted average 12-month 7-day / 30-day point prevalence cessation rate in previous telephone-based cessation interventions taking a proactive approach, which means that the quitting was not initiated by the smokers themselves, was 9% (ranging from 7 to 17%) [12]. While 9% is higher than the 4% success rate from quitting on one’s own, it is lower than the 14% weighted average success rate of telephone quit lines, where individuals initiate the effort to quit smoking [16, 17].

### Acceptance and commitment therapy (ACT) for smoking cessation

Acceptance and Commitment Therapy (ACT) is a psychological intervention that has been used for smoking cessations [18]. According to the study by Baker et al. [19], avoidance of distressing smoking-related thoughts, feelings, and bodily sensations is the principal motive for addictive behaviors such as smoking. The goal in ACT is to increase one’s acceptance of these aversive internal experiences by increasing psychological flexibility through targeting six major processes, namely “acceptance,” “defusion,” “self-as-context,” “the present moment,” “values,” and “committed action” [20]. In the context of smoking cessation, the acceptance component focuses on helping individuals to recognize and increase their willingness to experience the internal triggers of smoking without trying to control or avoid them, whereas the committed action component focuses on getting them to make commitments to engage in adaptive behavioral change guided by their values related to quitting [21].

Promising results have been found in several studies on the application of ACT to smoking cessation in general populations, namely, a greater abstinence rate than when the following therapies were used: Nicotine Replacement Therapy (NRT) [21]; Bupropion (front-line pharmacotherapy) [22], Cognitive Behavioral Therapy (CBT) [23, 24]; standard web-based smoking cessation interventions [25], and smartphone applications for smoking cessation [26]. To date, only three studies have been conducted on telephone-based ACT for smoking cessation: one with individuals with bipolar disorder [27] and two with general populations [24, 28]. These studies found that it is feasible to deliver ACT by phone, and that highly promising quit rates were obtained compared with standard CBT and quit line counseling. As these studies were conducted in the U.S. through a reactive approach (i.e., the participants were self-motived to quit smoking as they were recruited via Quitline or through an advertisement), it is unclear whether proactively recruiting individuals from primary care settings to an individual, telephone-based ACT would be more effective than a control intervention to achieve smoking cessation among a Chinese population, particularly among a Hong Kong (HK) Chinese population, as HK people tend to have busy lifestyles [29]. Rather than delivering ACT over an exclusively telephone-based approach, the ACT intervention was first initiated with a face-to-face meeting in a health care setting to increase the participants’ engagement in subsequent telephone sessions. The aim of this study was to determine the effectiveness of this ACT intervention approach as compared to the usual treatment at primary health care settings, by providing a very brief smoking cessation reminder and standardized self-help printed cessation materials, as well as to identify baseline predictors of self-reported quitting.

## Methods

### Study design and participants

The protocol for the present study has previously been described [30]. Briefly this study was a prospective, randomized trial with two parallel groups. Participants were recruited from six primary health care centers in Hong Kong. Attendees of the center were approached and smokers were identified through a preliminary screening with the help of staff of the clinic. Individuals who smoked were then invited to complete a baseline questionnaire and to undergo an exhaled carbon monoxide (CO) test administered by clinic staff or by the research assistants, who conducted the screening after the individuals had provided their written consent to be screened. Those who were interested in participating in the smoking cessation program and who met the criteria for inclusion were further invited to provide their written informed consent to take part in the study. The eligibility criteria were: (1) aged 18 years or above; (2) smoked at least one cigarette per day in the past 30 days; (3) not currently taking part in any other smoking cessation programs; (4) able to communicate in Cantonese; (5) a Hong Kong resident; (6) currently residing in Hong Kong and expecting to continue to do so for the next 6 months; and (7) have access to a telephone. The sample size calculations were based on the primary outcome of smoking cessation – the 7-day point prevalence quit rate as measured at the 12-month follow-up. Reference is made to the benchmark study by Bricker et al. [28] that used ACT by telephone approach where 29% of smokers quit smoking, compared with the 10% of smokers who quit with self-help materials [12]. To achieve a power of 80% at a 5% significance level for a chi-square test to detect a difference of 19% in the quit rate in 12 months (29% vs. 10%), a total of 136 subjects (68 per group) were required.

### Procedures

One hundred and forty-four individuals who met the criteria for inclusion and gave their written consent to participate in the study completed the baseline questionnaire. They were then randomized into either the intervention (ACT) group (*n* = 70) or the control group (*n* = 74). The process of randomization was based on computer-generated, block randomization with random block sizes, which were placed in sealed opaque envelopes. The randomization procedure was undertaken by another research assistant who was not directly involved in the study. The randomized study assignments were concealed from participants after eligibility were determined and consent to join this trial was obtained. Both groups received self-help materials on smoking cessation. Those in the ACT group also underwent an initial face-to-face session and two telephone ACT sessions at 1 week and 1 month following the first session. After 3, 6, and 12 months, they were re-contacted through telephone and were asked to complete the follow-up assessments of their smoking status, attempts to quit, and ability to deal with physical sensations, emotions, and thoughts without smoking. Those who reported as having quit at 6 and 12 months were then invited to undergo carbon monoxide (CO) and urinary cotinine tests. Participants of both groups also completed an evaluation survey through telephone at the 6- and 12-month follow-up sessions. From randomization through follow-up assessments, all research staff were blinded to the group allocations. The overall design of the study was illustrated in a published study protocol [30].

### Treatment conditions

#### Control and standard self-help materials

At the clinics, the participants were given a brief 5-min talk on the consequences of smoking. The benefits of quitting smoking were emphasized and the participants were advised to quit smoking. Written self-help materials describing strategies for tackling cravings, and containing information on community resources for quitting smoking were also provided. The printed materials were developed by Hong Kong’s Department of Health, and are available to the public [31].

#### Acceptance and commitment therapy (ACT)

In addition to the above self-help materials, participants in the ACT intervention were offered an initial, individual, face-to-face ACT session at the clinic and two telephone follow-up sessions at 1 week and 1 month following the initial ACT session. The sessions were delivered by an experienced health counselor trained in the principles of ACT applied in smoking cessation. Training was provided based on the ACT model developed by Hayes and colleagues [20]. Each session lasted around 15 to 20 min. A session-by-session ACT model for smoking cessation is comprised of six core processes, including “acceptance,” “defusion,” “self-as-context,” “the present moment,” “values,” and “committed action,” which work together to increase psychological flexibility. The acceptance component focuses on helping individuals to recognize and increase their willingness to experience the internal triggers of smoking, including physical sensations, feelings, and thoughts, without trying to control or avoid these experiences [30]. The ACT sessions used metaphors as well as mindfulness and experiential exercises to make it easier for the participants to identify their personal values in quitting smoking and taking committed actions towards the valued quitting goals. We selected ACT metaphors, such as the two sides of a coin metaphor that were relevant to Chinese people in the context of smoking and quitting. We then translated them into Chinese by the lead author (MYW) and one of the research staff (Mr Matthew Lee), both are bilingual in English and Chinese and experienced in conduct ACT intervention. The translations were validated by one of our team members (AYL) and clinical partners of the seven health clinics, who have extensive experience in helping smokers to quit smoking, and who are fluent in both English and Cantonese.

### Measures

The primary outcome was self-reported 7-day point prevalence abstinence at the 12-month follow-up session. Secondary outcomes included: (i) a readiness to quit smoking; (ii) the average number of cigarettes consumed daily in the past 12 months; (iii) the number of attempts to quit in the past 12 months; (iv-vi) the difficulty of quitting, confidence in quitting, and the importance of quitting; and (vii) the level of psychological flexibility.

#### Questionnaires

Information on the demographics of the participants and any of their smoking characteristics (e.g., smoking and quitting history and behaviors) known to predict smoking cessation [11] were recorded at baseline. Their stages of change, derived from Prochaska’s Transtheoretical Model, were measured by their readiness to quit smoking. There are five stages of change: pre-contemplation, contemplation, preparation, action, and maintenance [32]. The questionnaires also included the following scales:

- Seven-day Point Prevalence

This is a one-item question that assesses whether the participants have smoked in the past 7 days. Those who responded “no” are considered to have quit. This is the most common cessation outcome in smoking cessation trials [33].

- Fagerstrom Test for Nicotine Dependence (FTND)

The FTND is a six-item scale that assesses a person’s degree of nicotine dependence. It has adequate internal consistency and high test-retest reliability [34].

- Acceptance and Action Questionnaire-II (AAQ-II) for smoking

This 10-item scale is derived from an earlier version of the AAQ [35], which assesses the degree of experiential avoidance. The AAQ-II was developed to establish an internally consistent measure of the ACT model of mental health and behavioral effectiveness, and has strong psychometric properties with a mean alpha coefficient of 0.84(0.78–0.88). The 3- and 1-year test-retest reliabilities were found to be 0.81 and 0.79, respectively [36].

- Avoidance and Inflexibility Scale (AIS) [37]

The Avoidance and Inflexibility Scale (AIS) was derived as a smoking-specific measure of experiential avoidance [37, 38], while AAQ-II is the most widely used self-reported instrument for assessing the efficacy of ACT interventions [39]. This AIS scale was developed to measure the participants’ willingness to experience thoughts, feelings and sensations, and to not act on physical cravings to smoke. The AIS employs a five-point Likert scale, ranging from 1: not at all, to 5: very willing. The psychometric properties of the AIS have not been comprehensively evaluated among smokers. To the best of our knowledge, there are only two works, both unpublished, that have evaluated the psychometric properties of AIS [39, 40]. Cronbach’s alpha was used to assess the internal consistency of the AIS total score from baseline through to the 6-month follow-up session, with a Cronbach’s alpha score of 0.925–0.967 [39].

#### Biochemical measures

Exhaled CO and urinary cotinine are two common biochemical measures for validating self-reports of not smoking. Both have good sensitivity at 80–85% and 84–98%, and good specificity, at 90% + and 98%, respectively [33]. The level of exhaled CO was measured using a micro smokerlyzer CO monitor. Participants with exhaled CO levels of 6 ppm or above [41] or with a NicAlert test result at level 3 or above according to the manufacturer were considered to be current smokers.

#### Treatment fidelity

All ACT intervention sessions were audio-recorded and random samples of audio files (15–20%) were selected by two independent reviewers who are experienced in ACT using the ACT Core Competency Rating form [20]. They scrutinized the audio-recorded sessions for their completeness and adherence to the ACT intervention protocol. Discrepancies from the protocol that were identified were discussed with the counsellor for modification.

### Statistical analyses

Statistical analyses were carried out using the Statistical Package for the Social Sciences (SPSS) version 25.0. All tests were two tailed with a significance level of *p* < 0.05. An intention-to-treat (ITT) principle was applied to all the analyses whenever applicable. The participants’ demographics, smoking and quitting information, number of sessions attended, attrition rate, and satisfaction with the program were examined by means of descriptive statistics. Baseline data were analyzed to check the comparability of the intervention and control groups. Chi-square tests were performed for the categorical variables. Two-tailed *t-*tests were performed for continuous variables that were normally distributed and homoscedastic, while Mann-whiney U tests were performed for those were not, respectively. Primary evidence of the efficacy of ACT was assessed using risk ratio for the primary outcome (self-reported 7-day point prevalence abstinence at 12 months) and mean differences for the continuous secondary outcomes between the intervention and the control groups. Their associated 95% confidence intervals (CI) were also reported. A chi-square test was used to examine the differences between the two groups in stage of readiness to quit smoking at the 12-month follow-up session. Comparisons were also made between the two groups with regard to changes in the secondary outcomes (stage of readiness to quit smoking, importance of quitting, difficulty in quitting, confidence in quitting, and psychological flexibility) from baseline to the 12-month follow-up, using independent t-tests for continuous variables and chi-square tests for the categorical variables. Hochberg’s set-up procedure was used to control the overall type 1 error as 5% for testing 11 hypotheses in the study. Missing data were imputed by the last observation carried forward approach. Two sets of sensitivity analyses were performed with (1) missing data replaced by the baseline score, and (2) complete cases only. Significant variables in the bivariate analysis and treatment group were included as independent variables in the logistic regression model to identify the predictors of quitting smoking at 12 months. The goodness-of-fit for the model was assessed by the Hosmer-Lermershow test, where *p* > 0.5 indicates an agreeable model fit. The estimated adjusted odds ratios and 95% confidence intervals (CI) were reported.

## Results

### Sample attrition

**Fig. 1:**
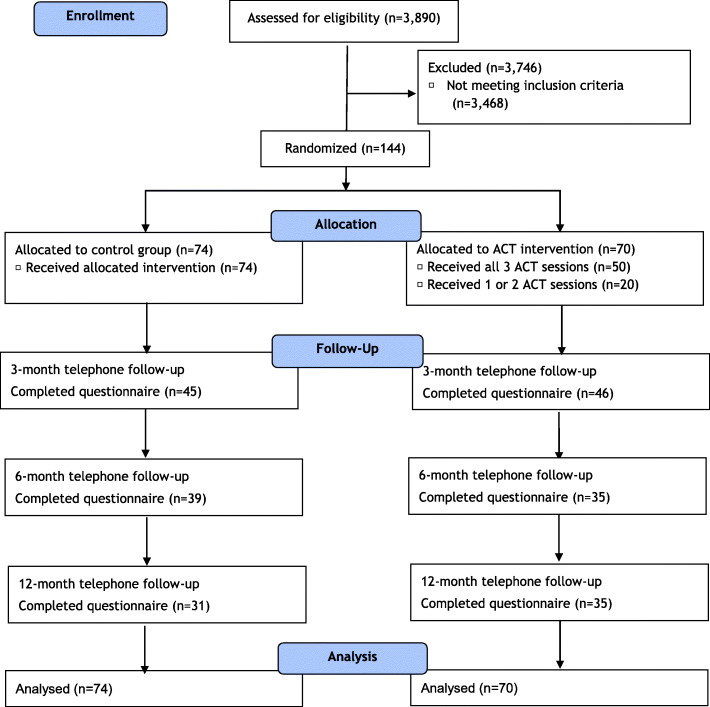
The flow of participants through the phases of the RCT

This study adheres to CONSORT guidelines and includes a completed CONSORT checklist as an addendum to this manuscript. Figure 1 shows the flow of participants through the phases of the RCT. A total of 3890 smokers were identified. Of the 423 smokers who agreed to complete the baseline questionnaires, 144 (34%) were eligible and agreed to be randomized into the study. The reasons given for not taking part in the study and the predictors of participation in the trial were reported eleswhere [42]. Seventy participants (48.6%) were allocated to the ACT group and 74 (51.4%) to the control group. There was no statistical significant difference in the retention rate between the two groups at the 3-month (intervention vs control: 65.7% vs 60.8%, *p* = 0.54), 6-month (50% vs 52.7%, *p* = 0.75) and 12-month follow-up (50% vs 41.9%, *p* = 0.33) respectively.

### Participants and randomization check

Table 1 shows the demographic and other baseline variables of the participants in each group. At baseline, one-fourth of the participants were female in the ACT group and one-third were female in the control group, while the mean age was 3 years older in the ACT group than in the contol group. The mean number of years of smoking was 19 years in the ACT group and 18 years in the control group. More than 70% of the participants smoked at home, and more than 60% in both groups had made a previous attempt to quit smoking. There were no significant differences between the intervention and control groups in any other sociodemographic variables, smoking-related variables, intention to quit, and perceived health status.

**Table 1:** Baseline Characteristics of the Participants

***Baseline variables***	Intervention (***N*** = 70)		Control (***N*** = 74)		***p***-value^‡^
	N	N(%)	N	N(%)	
**Sociodemographic Characteristics**					
Gender	70		74		0.279
Male		53 (75.7%)		50 (67.6%)	
Female		17 (24.3%)		24 (32.4%)	
Age	69	47.94 ± 14.37	74	44.85 ± 12.97	0.179^#^
Educational attainment	69		74		0.187
Primary or below		20 (29.0%)		12 (16.2%)	
Secondary or below		42 (60.9%)		53 (71.6%)	
Matriculation or above		7 (10.1%)		9 (12.2%)	
Marital Status	67		73		0.639
Single		12 (17.9%)		15 (20.5%)	
Married		51 (76.1%)		51 (69.9%)	
Divorced/ separated/widowed		4 (6.0%)		7 (9.6%)	
Employment status	68		74		0.182
Currently employed		53 (77.9%)		64 (86.5%)	
Unemployed		15 (22.1%)		10 (13.5%)	
Monthly household income	67		72		0.316
HK$9999 or less		11 (16.4%)		11 (15.3%)	
HK$10,000-29,999		34 (50.7%)		45 (62.5%)	
HK$30,000 or above		22 (32.8%)		16 (22.2%)	
Living with others	68		74		0.868
Yes		61 (89.7%)		67 (90.5%)	
No		7 (10.3%)		7 (9.5%)	
Had a partner who smoked	70		74		0.305
Yes		13 (18.6%)		19 (25.7%)	
No		57 (81.4%)		55 (74.3%)	
**Current smoking status and behavior**					
Smoking status	70		74		0.587
Daily smoker		69 (98.6%)		74 (100.0%)	
Occasional smoker		0 (0%)		0 (0%)	
Recently quit		1 (1.4%)		0 (0%)	
Average cigarette/day in the past 1 month	70		73		0.911
10 or fewer		25 (35.7%)		24 (32.9%)	
11–20		33 (47.1%)		37 (50.7%)	
21 or more		12 (17.1%)		12 (16.4%)	
Years of smoking	70	19.06 ± 6.61	74	18.10 ± 4.87	0.323^#^
Nicotine dependence level	70		74		0.383
Low		26 (37.1%)		35 (47.3%)	
Moderate		14 (20.0%)		15 (20.3%)	
High		30 (42.9%)		24 (32.4%)	
Smoke at home	70		74		0.194
Yes		54 (77.1%)		64 (86.5%)	
No		16 (22.9%)		10 (13.5%)	
Social support in quitting	70		74		1.000
Yes		67 (95.7%)		70 (94.6%)	
No		3 (4.3%)		4 (5.4%)	
**Quitting history**					
Previous attempt at quitting	70		74		0.419
No		22 (31.4%)		28 (37.8%)	
Yes		48 (68.6%)		46 (62.2%)	
**Intention to quit**					
Stages of change	70		74		0.227
Pre-contemplation		11 (15.7%)		16 (21.6%)	
Contemplation		26 (37.1%)		16 (21.6%)	
Preparation		31 (44.3%)		40 (54.1%)	
Action		2 (2.9%)		2 (2.7%)	

^‡^ p-value was obtained from a chi-square test

* Data presented as mean ± SD

^#^ *p*-value was obtained from Independent sample t- tests

### Intervention effects on smoking cessation outcomes

#### Primary outcome

The 7-day point-prevalence abstinence rates at the 12-month follow-up session were 24.3% (17/70) for the intervention group compared to 21.6% (16/74) for the control group. There was no significant difference in quitting status between the two groups (*p* = 0.704).

#### Secondary outcomes

Table 2 shows the significant differences between the two groups in the secondary outcomes using the last observation carried forward approach. Significantly more participants in the intervention group (21.4 and 8.6%) than in the control group (18.9 and 5.4%) expressed a readiness to quit smoking in the action and maintenance stages, respectively, with *p* = 0.003. The intervention group, who underwent ACT (mean ratings = 82.54, SD = 19.55), perceived that it was more important to quit than did the control group (mean ratings = 74.93, SD = 20.26), with *p* = 0.024. In addition, with regard to changes in the secondary outcomes from baseline to the 12-month follow-up, the participants in the intervention group demonstrated more improvement than those in the control group, in terms of the progress that they made in their readiness to quit smoking (41.4% vs. 27%, *p* = 0.014); and increased psychological flexibility (1.42 vs. 0.16 *p* = 0.022). However, only the between-group difference in stage of readiness to quit smoking at the 12-month follow-up was considered significant after controlling for multiple hypothesis tests. Tables 3 and 4 present the results of the two sensitivity analyses with missing data replaced respectively by baseline scores and complete data. Significant differences in stage of readiness to quit smoking and change in stages of readiness to quit smoking were obtained in the complete data analysis. More participants moved upward in the intervention group (Table 4) while none of the hypotheses were significant in the analysis when missing data were replaced by baseline scores (Table 3) when the overall type 1 error was controlled at 5%.

**Table 2:** Results on quit rate and readiness to quit smoking using the last observation carried forward approach

	Intervention (***N*** = 70)		Control (***N*** = 74)			
	N	n (%)	N	n (%)	Risk Ratio (95% CI)	***p***-value
**Primary outcome**						
Self-reported 7-day point prevalence abstinence at the 12-month follow-up	70	17 (24.3%)	74	16 (21.6%)	1.12 (0.62–2.05)	0.704
Bio-validated quit rate					0.63 (0.16, 2.56)	0.261
**Secondary outcomes**						
Readiness to quit smoking	70		74			0.003*
Pre-contemplation		11 (18.6%)		35 (47.3%)		
Contemplation		14 (18.6%)		11 (14.9%)		
Preparation		29 (32.9%)		10 (13.5%)		
Action		10 (21.4%)		14 (18.9%)		
Maintenance		6 (8.6%)		4 (5.4%)		
		**Mean ± SD**		**Mean ± SD**	**Mean Difference (95% CI)**	
Average daily cigarette consumption in the past 12 months	70	11.22 ± 9.24	73	11.17 ± 8.43	0.052 (−2.87–2.97)	0.972
Average number of quit attempts in the past 12 months	70	0.79 ± 1.19	74	0.66 ± 1.82	0.13 (− 0.38–0.63)	0.632
Importance of quitting	69	82.54 ± 19.55	74	74.93 ± 20.26	7.60 (1.01–14.20)	0.024
Difficulty in quitting	69	69.28 ± 23.16	74	64.39 ± 27.93	4.88 (−3.59–13.35)	0.256
Confidence in being able to quit	69	63.33 ± 23.93	73	58.29 ± 27.19	4.32 (− 4.14–12.78)	0.312
Psychological flexibility	60	27.80 ± 3.52	53	27.42 ± 3.68	0.38 (− 0.96–1.73)	0.571
**Changes in the secondary outcomes from baseline to 12 months**						
		**n (%)**		**n (%)**		
Readiness to quit smoking	70		74			0.014
Less than before		13 (18.6%)		30 (40.5%)		
Same as before		28 (40.0%)		24 (32.4%)		
More than before		29 (41.4%)		20 (27.0%)		
		**Mean ± SD**		**Mean ± SD**	**Mean Difference (95% CI)**	
Average number of quit attempts	70	0.79 ± 1.19	74	0.66 ± 1.82	0.13 (− 0.38–0.63)	0.632
Importance of quitting	69	10.22 ± 19.41	74	4.93 ± 17.95	5.28 (− 0.89–11.46)	0.093
Difficulty in quitting	69	− 1.38 ± 16.13	74	− 1.42 ± 14.70	0.04 (− 5.06–5.14)	0.987
Confidence in being able to quit	69	9.13 ± 20.49	73	2.19 ± 20.09	6.21 (− 0.73–13.16)	0.079
Psychological flexibility	48	1.42 ± 2.94	44	0.16 ± 2.22	1.26 (0.18–2.33)	0.022

* Statistically significant at an overall type 1 error of 5% based on Hochberg’s set-up procedure

**Table 3:** Results on quit rate and readiness to quit smoking using imputation of baseline data

	Intervention (***N*** = 70)		Control (***N*** = 74)			
	N	n (%)	N	n (%)	Risk Ratio (95% CI)	p-value
**Primary outcome**						
Self-reported 7-day point prevalence abstinence at the 12-month follow-up	70	12 (17.1%)	74	10 (13.5%)	1.27 (0.59–2.75)	0.273
Bio-validated quit rate					0.63 (0.16, 2.56)	0.261
**Secondary outcomes**						
Readiness to quit smoking					**–**	0.054
Pre-contemplation	70	11 (15.7%)	74	27 (36.5%)		
Contemplation		14 (20.0%)		12 (16.2%)		
Preparation		29 (41.4%)		25 (33.8%)		
Action		10 (14.3%)		8 (10.8%)		
Maintenance		6 (8.6%)		2 (2.7%)		
		**Mean ± SD**		**Mean ± SD**	**Mean Difference (95% CI)**	
Average daily cigarette consumption in the past 12 months	70	13.04 ± 9.37	73	13.88 ± 8.65	− 0.85 (− 3.83–2.13)	0.575
Average number of quit attempts in the past 12 months	70	1.66 ± 1.67	74	1.74 ± 1.78	− 0.08 (− 0.65–0.49)	0.785
Importance of quitting	69	79.93 ± 20.73	74	73.85 ± 20.57	6.08 (− 0.75–12.91)	0.081
Difficulty in quitting	69	68.41 ± 23.24	74	65.61 ± 27.98	2.80 (− 5.69–11.28)	0.515
Confidence in being able to quit	69	60.29 ± 24.25	73	58.97 ± 26.42	1.32 (−7.11–9.75)	0.758
Psychological flexibility	60	27.88 ± 3.52	53	27.42 ± 3.62	0.46 (− 0.88–1.81)	0.495
**Changes in the secondary outcomes from baseline to 12 months**						
		**n (%)**		**n (%)**		
Readiness to quit smoking	70		74		–	0.014
Less than before		7 (10.0%)		19 (25.7%)		
Same as before		43 (61.4%)		46 (62.2%)		
More than before		20 (28.6%)		9 (12.2%)		
		**Mean ± SD**		**Mean ± SD**	**Mean Difference (95% CI)**	
Importance of quitting	69	7.61 ± 18.64	74	3.85 ± 15,018	3.76 (− 1.89–9.41)	0.190
Difficulty in quitting	69	−2.25 ± 14.18	74	−0.20 ± 13.25	− 2.04 (− 6.58–2.49)	0.375
Confidence in being able to quit	69	6.09 ± 19.34	73	2.88 ± 16.03	3.21 (− 2.70–9.13)	0.285
Psychological flexibility	48	1.42 ± 2.93	44	0.16 ± 2.00	1.26 (0.22–2.29)	0.018

**Table 4:** Results on quit rate and readiness to quit smoking using complete data

	Intervention (***N*** = 35)		Control (***N*** = 31)			
	N	n (%)	N	n (%)	Risk Ratio (95% CI)	***p***-value
**Primary outcome**						
Self-reported 7-day point prevalence abstinence at the 12-month follow-up	70	12 (17.1%)	74	10 (13.5%)	1.06 (0.54–2.11)	0.174
Bio-validated quit rate					0.53 (0.14, 2.04)	0.179
**Secondary outcome**						
Readiness to quit smoking					**–**	0.001*
Pre-contemplation	70	7 (20.0%)	74	17 (54.8%)		
Contemplation		2 (5.7%)		5 (16.1%)		
Preparation		12 (34.3%)		0 (0%)		
Action		8 (22.9%)		7 (22.6%)		
Maintenance		6 (17.1%)		2 (6.5%)		
		**Mean ± SD**		**Mean ± SD**	**Mean Difference (95% CI)**	
Average daily cigarette consumption in the past 12 months	34	8.43 ± 9.45	31	9.50 ± 7.58	−1.07 (−5.35–3.20)	0.617
Average number of quit attempts in the past 12 months	32	1.69 ± 0.47	28	1.54 ± 0.51	0.15 (−0.10–0.41)	0.235
Importance of quitting	35	87.86 ± 18.36	31	77.26 ± 18.25	10.60 (1.58–19.62)	0.022
Difficulty in quitting	35	69.14 ± 25.25	31	60.48 ± 26.59	8.66 (−4.15–21.46)	0.180
Confidence in being able to quit	35	68.00 ± 26.32	31	64.84 ± 25.93	3.16 (−9.72–16.04)	0.625
Psychological flexibility	35	28.97 ± 3.12	25	27.64 ± 3.89	1.33 (− 0.48–3.15)	0.147
**Changes in the secondary outcomes from baseline to 12 months**						
		**n (%)**		**n (%)**		
Readiness to quit smoking	35		31		–	0.003*
Less than before		7 (20.0%)		19 (61.3%)		
Same as before		8 (22.9%)		3 (9.7%)		
More than before		20 (57.1%)		9 (29.0%)		
		**Mean ± SD**		**Mean ± SD**	**Mean Difference (95% CI)**	
Importance of quitting	35	15.00 ± 24.10	31	7.90 ± 24.21	7.10 (−4.80–19.00)	0.238
Difficulty in quitting	35	−4.43 ± 19.81	31	−0.48 ± 20.67	−3.94 (−13.91–6.02)	0.432
Confidence in being able to quit	35	12.00 ± 25.99	31	6.77 ± 24.27	5.23 (−7.19–17.64)	0.404
Psychological flexibility	25	2.72 ± 3.63	16	0.43 ± 3.37	2.28 (− 0.01–4.57)	0.051

* Statistically significant at an overall type 1 error of 5% based on Hochberg’s set-up procedure

### Predictors of quitting

In Table 5, a multiple logistic regression analysis was performed to test all of the above significant variables. Several independent variables were found that significantly contributed to success at quitting smoking. They were: age (*p* < 0.01), monthly household income (*p* < 0.01), number of cigarettes consumed in the past month at the baseline assessment, and those who perceived themselves to be more confident about quitting (*p* < 0.001). Participants who underwent ACT were 96% more likely to quit smoking than those who had been allocated to the control group. However, this effect was not statistically significant.

**Table 5:** Logistic regression of significant predictors on the primary outcome at the 12-month follow-up session

	Self-reported seven-day point prevalence abstinence at the 12-month follow up (No vs. Yes)		
	Adjusted OR	95% CI	*p*-value
Age			
18–35 (Young adults)	0.57	0.14–2.30	0.427
36–55 (Middle-aged)	0.13	0.031–0.54	0.005
56 or above (Older adults) (referent)	1		
Monthly household income			
HK$9999 or less (referent)	1		
HK$10,000-29,999	0.12	0.026–0.53	0.005
HK$30,000 or above	0.26	0.047–1.42	0.120
Average cigarette/day in past 1 month			
10 or less	0.33	0.061–1.83	0.206
11–20	0.099	0.016–0.63	0.014
21 or above (referent)	1		
Difficulty in quitting	0.98	0.95–1.00	0.078
Confidence in being able to quit	1.06	1.03–1.10	< 0.001
Mental Component Summary (MCS)	1.05	0.98–1.12	0.158
Group			
Intervention	1.96	0.63–6.10	0.244
Control (referent)	1		

### Treatment Fidelity and acceptability

An ACT counselor conducted all of the ACT sessions. This counselor is a professional member of the Association of Contextual Behavioral Science (ACBS) and has worked as a private ACT practitioner helping people with psychological difficulties or substance misuse. Throughout the study, his ACT skills were supervised by the principal investigator of the study and the first author of this report (YWM), a professional member of the ACBS and experienced ACT researcher. After each ACT session, the counselor completed the ACT competency checklist [20]. In addition, all completed ACT sessions of 18 out of 70 participants from the ACT group (the assessment rate was 24%) were audio-recorded and evaluated independently by YWM and another peer counselor. Fidelity findings indicated that the ACT counselor generally adhered well to the principles of ACT. The well-performed items (80–90% adhered to the principles – very satisfactory) were therapeutic stance, undermining cognitive defusion, and building patterns of committed action. Two principles were adhered to at a moderately satisfactory level, at between 60 and 70%. The two items were: used the concept of “workability” in interacting with the participants, and used appropriate exercises or metaphors to show willingness (acceptance/openness) as an effective alternative to avoiding difficult internal experiences or helping the participants notice cravings and urges (thoughts, feelings, and bodily sensations), while also contacting a self who chooses and behaves with these experiences, rather than for these experiences. During their weekly meetings, the counselor, YWM, and the peer counselor discussed any noteworthy discrepancies between the ACT protocol and the ACT sessions, and worked out strategies on how to adhere to protocol in the coming sessions.

Of the 70 participants who joined the intervention group, 50 (71.4%) completed all three sessions. On a scale of 1 (did not practice at all) to 5 (had regular practice), the participants in the intervention group were asked to rate their regularity in practicing or applying the techniques that they had learned for dealing with smoking cravings; the mean rating given by the ten participants who responded to this item was 2.20 (SD = 1.23).

## Discussion

To the authors’ best knowledge, this is the first study to evaluate the effectiveness of an individual ACT for smoking cessation delivered initially in a primary health care setting and subsequently by telephone in a Chinese population. The results suggest that there was no significant difference between the ACT and the control group in the 7-day point-prevalence abstinence rates at the 12-month follow-up, but significant outcomes were observed in the intervention group when compared with the control group from baseline to the 12-month follow-up. These included a forward progression in the participants’ readiness to quit smoking, increased confidence in quitting smoking, and increased psychological flexibility. It is noteworthy that the quality of the evidence in the present study is high, as assessed using a standard protocol consisting of a randomized controlled trial with good intervention fidelity. However only marginal benefits were observed; therefore it might be insufficient to use a single smoking cessation intervention (ACT) to help smokers to quit smoking.

The ACT and control groups did not differ in quitting status. Individuals were recruited from seven Health Maintenance clinics, where individuals are more motivated to take action to maintain their health, which might explain the unexpectedly high quitting rate (21.6%) in the control group despite receiving self-help materials only, as compared to previous studies (2–10%) [7, 43]. Another possible explanation is that individuals in the control group might have utilized the community resources for quitting smoking that were mentioned in the written self-help materials provided to them. In addition, the insignificant difference between the two groups might be due to the brevity of the intervention in this study and the lack of practice in ACT techniques as reported by participants in the ACT group, such as being mindful of the moments when one has cravings to smoke or the related withdrawal symptoms, while also persisting in quitting smoking in a way that aligns with one’s personal values. The quit rate (i.e., 7-day point prevalence) in the ACT group in this study was difficult to compare to that in the first trial of a 5-session, 90-min telephone-delivered ACT together with NRT, as a 30-day point prevalence abstinence rate (29%) was utilized in that study [28].

The participants in the ACT group were found to be cognitively more ready to quit smoking in terms of their psychological flexibility, readiness in quitting and confidence in quitting from baseline to the 12-month follow-up, as compared to the control group. However, the changes were not reflected in behavioral measures (e.g., the number of cigarettes consumed and the number of quit attempts). It is worth noting that, based on recent research results [44], it is recommended that the stage of change model is uninformative to predict the chance of successful smoking cessation.” Thus, it is quite clear that there have no benefit to smoking cessation with application of the current intervention. From baseline to the 12-month follow-up, the participants in the ACT group were significantly more confident than those in the control group that they could quit smoking. This finding is consistent with a study that utilized ACT for smoking cessation among veterans with Post-traumatic Stress Disorder, which also found that ACT increased the participants’ confidence in quitting [45]. Increased self-efficacy in resisting smoking was identified as a significant mediator of the effectiveness of smoking cessation treatments [28, 46, 47].

Participants in the ACT group showed significantly greater reported psychological flexibility when dealing with cravings compared to the control group, as measured by the smoking-specific AAQ, suggesting that ACT helped the participants to respond more flexibly to negative internal experiences including urges to smoke. The finding of increased psychological flexibility following ACT is consistent with that of previous studies, where acceptance of physical cravings, emotions, and smoking-related thoughts as measured by the Avoidance and Inflexibility Scale (AIS) increased following telephone-delivered ACT for smoking cessation [24, 27, 28]. The increase in psychological flexibility following ACT is clinically meaningful, as previous studies found that increasing psychological flexibility serves as an essential element in smoking cessation treatments [21, 22, 25]. However, significantly greater psychological flexibility in the ACT group did not translate to a significantly greater quit rate relative to the control group. Thus, the future studies may use a combination of smoking cessation medications and a behavioral intervention to yield better quit rates when compared with using behavioral interventions only.

The study retention rate at the 6-month follow-up in this study (51.3%) was slightly lower than a previous study utilizing a telephone-delivered ACT for smoking cessation with a general population of smokers (66% at the 6-month follow-up) [24], but was comparable to the average study retention rate of 55% in comparable telephone-delivered smoking cessation interventions for general populations in the West [48–50]. This suggests that the intervention in this study was feasible in terms of retaining participants among a Chinese population. However, the participants in the ACT group seldom practiced the ACT skills to deal with their cravings, suggesting that they took on a more passive role. It is also possible that they had not achieved complete competence in applying the techniques taught in the brief sessions. A future study may benefit from adjustments such as providing the participants with longer, more intensive instructions and conducting a more thorough evaluation of the participants’ understanding and use of acceptance as a technique for smoking cessation.

The current study has several limitations. First, with a retention rate of far less than 100% it is difficult to know the actual quit rates for each arm. Caution should be exercised when interpreting the findings. Second, no information was obtained in this study on how the participants responded to the components of the ACT and which components were more effective. Yet every effort was made to ensure the fidelity and quality of the intervention delivered in this study. With regard to future research, the role of counseling techniques as agents of treatment outcomes should be examined, as the data suggested that ACT-specific techniques (e.g., awareness, openness, etc.) were to some degree used as predictors of smoking probabilities in the following session [51]. This will advance our understanding of counselor-level processes of change in ACT for smoking cessation. Third, more research is needed on the mechanisms underlying ACT-based effects for smoking cessation. ACT treatment targets processes beyond those of experiential avoidance and flexibility (e.g., values, commitment to quitting). Therefore, there is a need to explore the explanatory relevance of other ACT components in smoking cessation and to empirically evaluate if and how they contribute to smoking outcomes. Future research may also include ACT-consistent secondary outcome measures such as quality of life, psychosocial functioning, and values-consistent living. Fundamentally, the goal of ACT is not simply to increase the quit rate, but to improve overall levels of functioning (e.g., living a meaningful, valued life). While a reduction in smoking might ultimately lead to improved functioning, it should not be the only focus of treatment, and therefore should not be the only measure of the success of a treatment. Last, but not least, stages of change should be abandoned to use in assessing smoking cessation outcomes since it is uninformative [44].

## Conclusions

The findings suggest that the ACT intervention can feasibly be applied in natural clinical settings and could motivate smokers to become more open to quitting smoking. However, three brief sessions of ACT are not produce benefit to help them to quit smoking. Nevertheless, this study provides a number of key advances over the current evidence on ACT for smoking cessation. Specifically, this study provides the first evidence of an RCT on the adoption of an individual ACT for smoking cessation, delivered initially in primary health care settings and subsequently by telephone within a Chinese population in a briefer format than in previous studies. Overall, the results suggest that ACT for smoking cessation brought about cognitive changes, including greater psychological flexibility, and more confidence about quitting, when compared to the use of self-help materials only among the general population in Hong Kong. The effect of an ACT intervention combined with other interventions could be investigated in the future studies, which may help to translate these cognitive changes into smoking abstinence.

## Abbreviations

AAQ-II: Acceptance and Action Questionnaire-II
ACT: Acceptance and Commitment Therapy
AIS: Avoidance and Inflexibility Scale
CBT: Cognitive Behavioral Therapy
CO: Carbon monoxide
FTND: Fagerstrom Test for Nicotine Dependence
HK: Hong Kong
ITT: Intention-to-treat
NRT: Nicotine Replacement Therapy
RCT: Randomized controlled trial
SPSS: Statistical Package for the Social Sciences
